# Commentary: The Value of Failure in Science: The Story of Grandmother Cells in Neuroscience

**DOI:** 10.3389/fnins.2020.00059

**Published:** 2020-02-05

**Authors:** Asim Roy

**Affiliations:** Department of Information Systems, Arizona State University, Tempe, AZ, United States

**Keywords:** concept cells, grandmother cells, multisensory neurons, abstract concept, multimodal invariance, sparse coding, associative learning

This commentary relates to the *Frontiers in Neuroscience* article “*The Value of Failure in Science: The Story of Grandmother Cells in Neuroscience*” by Ann-Sophie Barwich. This commentary is not against the idea of failure analysis, but about the argument that grandmother cells is a failed notion. I summarize first the discussion in this article by Barwich about grandmother cells (and related concepts—concept cells, associative learning, hierarchical processing, sparse coding, and so on) in the sequence they were presented (all references are in the Barwich article): (1) that they respond to a specific but complex stimulus; (2) that they account for selectivity and specificity of neural responses and localization and convergence in neural hierarchical processing; (3) that localization as a paradigm continues to persist; (4) that according to Marr (1982), answers about mind and brain could not be found at the single cell level; (5) that the framework of convergent, hierarchical, and localized signal integration has not been successful; (6) however, even after this general characterization, it's still unclear to Barwich what grandmother cells actually were; specifically, to what processes they referred to; (7) that grandmother cells were opaque both on a theoretical and a practical level; (8) that it is undetermined what kind of information was processed and integrated with such a hypothetical neuron; do they only respond to visual input or also process cross-modal cues including auditory and olfactory signals; if they do cross-modal integration, where is the centrum of such integration; (9) that the broader question is how does unified phenomenal experience arise from separated and specialized neural processes; (10) that the scope of the grandmother cell concept was not clear-cut; do they respond only to a particular individual (a grandmother) or to a category (grandmothers); (11) she then introduces the notion of concept cells through Konorski's gnostic units, which were associated with recordings of localized responses to complex objects, such as faces; (12) argues that although concept and grandmother cells and gnostic units seemed sufficiently similar, they are not because they have different processes; (13) then she illustrates concept cells (Quiroga et al., 2008)—single neurons that respond selectively to various visual representations of individual objects (Sydney Opera House) and people (Jennifer Aniston); (14) then introduces sparse coding (activation of small groups of neurons) as an explanation for specialized neural responses (e.g., faces); (15) argues that sparse coding is not exclusive to a specific stimuli, such as Halle Berry; (16) that sparse coding is a different theory of neural representation—sets of neurons build a net of neural representation, potentially unrelated in their feature coding pathways (e.g., visual input integrated with auditory input); that these sets of neurons do not code concepts strictly bottom-up from simple signals to complex individuals and categories; (17) that the main difference between grandmother cells and sparse coding is that grandmother cells emerged from the hierarchical coding hypothesis, but sparse coding doesn't require hierarchical coding; (18) that sparse coding uses a separate theory of learning, that of associative learning; (19) and claims that it's debatable whether abstraction defines the ultimate nature of the brain.

Commentary - There are many flawed arguments in the analysis presented by Barwich as summarized above. Here are some of the main ones.

1. The claim that grandmother cells do not use associative learning across modalities is false:
  Gross (2002) states that grandmother cells were conceived as “multimodal” and Roy's (2013) “multimodal invariant” characterization is consistent with Gross. Reddy and Thorpe (2014) also define concepts cells as “invariant, multimodal.” Thus, the fundamental characterization of these two concepts is identical. “Multimodal invariance” implies associative learning across modalities.
  The claim by Barwich that concept cells use associative learning whereas grandmother cells do not or that they have different learning processes is false.
2. If a neuron's activity has “meaning and interpretation,” it cannot be part of sparse population coding:
  Sparse coding is a form of population coding that uses a limited set of neurons. By definition, in population coding, one cannot assign “meaning and interpretation” to the activity of a single neuron. Reddy and Thorpe (2014) state that concept cells have “***meaning*** of a given stimulus in a manner that is ***invariant*** to different representations of that stimulus.” Since one can assign “meaning” to the activation of a concept cell, then that cell, theoretically, could not be part of any population coding scheme. It would be a contradiction. Thus, Barwich falsely claims that concept cells are part of a sparse population coding scheme.
3. Grandmother and concept cells are the same; any argument against one also applies to the other:
  “**Selectivity or specificity**,” “**complex concept**,” “**meaning**,” “**multimodal invariance**” and “**abstractness**” (Reddy and Thorpe, 2014) are all integral properties of both concept cells and grandmother cells. Barwich argues that they are different concepts. That's because Barwich never rigorously compares the definition of concept cells by Reddy and Thorpe (2014) with that of grandmother cells. With a rigorous comparison, Barwich certainly would have realized that the whole narrative against grandmother cells applies to concept cells as well.
4. Barwich denies single cell abstractions in the brain despite the neurophysiological evidence:
  Barwich also denies the existence of single cell abstractions although Reddy and Thorpe (2014) categorically state that “***abstract, invariant representations***” is “*a hallmark of MTL concept cells*.” Quiroga (2012) also characterize concept cells as abstract: “*These and many other examples suggest that MTL neurons encode an* ***abstract*** representation of the concept triggered by the stimulus.” Quiroga et al. (2008) estimate that 40% of MTL cells are abstract. Therefore, Roy's (2017) claim that the brain uses a single-cell based abstract cognitive system is well-supported by neurophysiological evidence.

Overall, Barwich's analysis lacks rigorous comparison of ideas, uses flawed arguments and has false claims. In addition, characterizations of concept cells by Reddy and Thorpe (2014) and others confirm the prediction of Barlow (2009) that grandmother cells exist and “*can now be recorded from and studied reliably*.” One should also note that grandmother cells are just a special type of complex abstract cell. Multisensory neurons provide extensive evidence for other types of abstract cells in the brain (Roy, 2017).

## Author Contributions

The author confirms being the sole contributor of this work and has approved it for publication.

### Conflict of Interest

The author declares that the research was conducted in the absence of any commercial or financial relationships that could be construed as a potential conflict of interest.
